# Authors’ response

**DOI:** 10.1308/003588412X13373405386376

**Published:** 2012-09

**Authors:** T McBride

**Affiliations:** Sandwell and West Birmingham Hospitals NHS Trust, West Bromwich,UK

The audit undertaken by Weston *et al* highlights very nicely the lack of emphasis on spending time talking to patients and relatives regarding the serious nature of hip fractures. Since publishing my work on the public perceptions of hip fractures I have endeavoured to make time for such explanation when dealing with this patient group, especially in those patients who are cognitively impaired. I have also made it a priority to attempt to educate junior orthopaedic staff on this matter.

It still surprises me that most hospital trusts do not include written information on hip fractures within the care pathway. A section or tick box in the pathway to document discussion on this matter with the patient and relatives would also be helpful.

